# Plastic Surgery

**Published:** 1890-06

**Authors:** H. H. Spiers

**Affiliations:** Edinburg, O.


### Plastic Surgery.

BY H. H. SPIERS, M.D., EDINBURG, O.

In the summer of 1882 Mr. H., aged fifty, while attending a shingle machine, attempted to pick from the block, or stand, a piece of shingle while the knife was running. Result: Two inches lost from each of two middle fingers. I saw the patient about two hours after the injury. The cut was horizontal, and the stumps were ligated by having a strong twine tied around them. I inquired for the severed pieces. On saying I wished to replace them the laugh was general at my experimental surgery.

A boy was sent to get the amputated pieces from the machine, about forty rods distant. He was afraid to touch them, so they were carried on a shingle. In coming back he stumbled and lost one of the pieces in the grass. It could not be found. The one brought was replaced and retained *in situ* by adhesive strips. At the patient's request the other stump was left as amputated, dressed with balsam fir. In one week the replaced member was very much discolored; no odor or discharge. In two or three weeks the dark cuticle came off and the finger became red and sensitive. In five or six weeks recovery was complete except stiffness of the third joint. One year later the finger was as useful as any of the others, but was always sensitive to extreme cold. The amputated finger is, of course, out of the way, but is very clumsy and of little use. On asking the question, Which of the fingers do you prefer? the reply is: "I much prefer the replaced one."

In August, 1885, I reported five cases of accidental amputation, including this one, to the Portage County Medical Society. I trust, in the near future, to be able to present some rules for guidance in these and similar cases.

